# Vaccine-Associated Anterior Uveitis

**DOI:** 10.3390/vaccines14080672

**Published:** 2026-08-02

**Authors:** Seyyedehfatemeh Ghalibafan, Mona Oraei, Mohammadali Ashraf, Ali R. Djalilian, Pooja Bhat, Hajirah N. Saeed

**Affiliations:** 1 Department of Ophthalmology and Visual Sciences, University of Illinois Chicago, Chicago, IL 60612, USA; fatima25@uic.edu (S.G.); moraei@uic.edu (M.O.); mashra7@uic.edu (M.A.); adjalili@uic.edu (A.R.D.); pbhat@uic.edu (P.B.); 2 Department of Ophthalmology, Loyola University Medical Center, Maywood, IL 60153, USA

**Keywords:** vaccine-associated anterior uveitis, anterior uveitis, uveitis, iridocyclitis, vaccine safety, ocular inflammation, vaccination, immunization, adverse events, molecular mimicry

## Abstract

Vaccine-associated anterior uveitis (VAAU) is an uncommon ocular inflammatory phenotype reported after several vaccine platforms. It describes anterior segment inflammation occurring after vaccination when infectious, autoimmune, idiopathic, medication-related, and other possible causes have been reasonably excluded. The current literature indicates that post-vaccination uveitis is rare relative to the scale of immunization, although anterior uveitis is frequently reported among post-vaccination ocular inflammatory events. Evidence remains limited by reliance on case reports, case series, retrospective cohorts, and passive surveillance data, which restricts precise incidence estimation and causal interpretation. Accordingly, the available evidence primarily supports a temporal association rather than a confirmed causal relationship. Proposed mechanisms include innate immune activation, molecular mimicry, adjuvant- or formulation-related immune stimulation, bystander activation, delayed-type hypersensitivity, and host susceptibility related to prior uveitis, autoimmune disease, immune dysregulation, or genetic predisposition. Clinically, VAAU may present with redness, pain, photophobia, blurred vision, anterior chamber cells and flare, keratic precipitates, posterior synechiae, elevated intraocular pressure, vitritis, or cystoid macular edema. Most cases described in the literature are mild to moderate and improve with topical corticosteroids and cycloplegic therapy, whereas recurrent, severe, or treatment-intensive courses have been reported mainly in predisposed individuals. Future work should emphasize active surveillance, standardized phenotype-specific reporting, harmonized case definitions, and mechanistic studies to better define risk, recurrence patterns, and causality. Careful interpretation of suspected cases can support accurate diagnosis, patient counseling, and vaccine-safety monitoring without undermining clinically indicated immunization.

## 1. Introduction

Vaccination has been among the most successful achievements of preventive medicine, substantially reducing the global burden of infectious disease and enabling rapid public-health responses to emerging pathogens [1]. Continued advances in vaccine design have expanded available immunization platforms, including mRNA, viral vector, recombinant protein, inactivated, and live attenuated vaccines [2,3]. As immunization programs continue to expand across diverse populations, careful recognition and interpretation of adverse events following vaccination remain essential for clinical practice and vaccine safety surveillance [3,4].

Vaccine-associated anterior uveitis (VAAU) refers to anterior-segment intraocular inflammation temporally associated with vaccination, after exclusion of more common infectious, autoimmune, idiopathic, medication-related, and other possible etiologies [5,6,7,8]. Given the frequency and etiologic diversity of anterior uveitis in ophthalmic practice, suspected VAAU should not be attributed to immunization based on timing alone [6,7,8].

The coronavirus disease 2019 (COVID-19) vaccination era renewed interest in post-vaccination ocular inflammation amid the unprecedented scale of immunization and increased reporting of rare adverse events [8,9,10,11,12]. However, many reports remain difficult to interpret because anterior uveitis is often embedded within broader post-vaccination uveitis datasets and passive surveillance systems [8,9,10,11,12]. These limitations complicate phenotype-specific risk estimation and causal attribution [8,9,10,11,12].

In this review, we aim to evaluate the available evidence on VAAU and provide a practical framework for diagnosis, management, patient counseling, and vaccine-safety interpretation. To the best of our knowledge, this is the first review dedicated specifically to VAAU as a distinct anterior-segment inflammatory entity, integrating phenotype-specific evidence with considerations of causality and clinical decision-making.

## 2. Epidemiology and Evidence Base

### 2.1. Scope of Available Evidence

The epidemiology of VAAU remains difficult to define because many studies assess vaccine-associated uveitis as a broad outcome without consistently stratifying cases by anatomic subtype, including anterior, intermediate, posterior, or panuveitic involvement [8,10]. As a result, anterior uveitis-specific evidence is often derived from subgroup analyses, phenotype-level descriptions, and mixed clinical or pharmacovigilance cohorts rather than from studies designed specifically to evaluate VAAU [8,10,13].

### 2.2. Incidence Estimates and Reporting Patterns

Available evidence suggests that post-vaccination uveitis is uncommon relative to the scale of vaccine administration, although anterior uveitis is frequently reported among ocular inflammatory phenotypes [8,11]. Padhi et al. conducted a systematic review and meta-analysis of six retrospective observational studies including data from more than 2 billion administered vaccine doses and found a pooled post-COVID-19 vaccination uveitis incidence of 0.016% (95% CI, 0.010–0.026) [14]. Broader systematic reviews of reported ocular adverse events similarly identify uveitis as a prominent category of post-vaccination ocular inflammation [5,15]. Zou et al. identified uveitis as the most frequently reported ocular event after non-COVID antiviral vaccination, accounting for 92 of 184 affected eyes (50.0%; 95% CI, 42.8–57.2%) [5]. Ebrahimi et al. reviewed 76 eligible studies describing 107 reported ocular complications following COVID-19 vaccination and identified uveitis as the leading reported complication [15]. While these studies did not specifically evaluate anterior uveitis, and it was not possible to discern this from the available data, in a multinational case series of 70 patients, Testi et al. reported that anterior uveitis was the dominant ocular inflammatory phenotype after COVID-19 vaccination, accounting for 41 cases (58.6%) [11].

Controlled epidemiologic studies provide more direct risk assessment but have yielded mixed findings. Shemer et al. found no statistically significant association between BNT162b2 vaccination and new-onset acute anterior uveitis in a matched case–control study (OR, 0.508; *p* = 0.300) [16]. Consistent with this finding, the overall number of acute anterior uveitis cases observed during the 2021 vaccination period did not appear higher than that reported during the corresponding months of the preceding 6 years [16]. In contrast, Tomkins-Netzer et al. reported a small population-level increase in active noninfectious uveitis after BNT162b2 vaccination, with a low attributable risk and anterior uveitis accounting for most cases; however, the authors emphasized that these findings did not establish causality [17]. Kumar et al. found no increased overall risk of noninfectious uveitis after COVID-19 vaccination among individuals without prior uveitis, although a signal in younger individuals required further study [18]. Similarly, Kim et al. found no increase in recurrent uveitis after COVID-19 vaccination in a nationwide matched cohort and crossover case-series study; in self-controlled analyses, uveitis occurred at similar frequencies before and after vaccination despite anterior uveitis comprising 84.5% of prior uveitis cases [19]. In contrast, J. Kim et al. reported an increased recurrence risk in a nationwide cohort of 473,934 vaccinated individuals with prior uveitis, with cumulative post-vaccination uveitis rates of 8.6% at 3 months and 16.8% at 1 year, predominantly involving anterior uveitis [20].

Large-scale pharmacovigilance and regulatory datasets provide complementary reporting signals. Oh et al. analyzed reports from the World Health Organization (WHO) VigiBase, the global pharmacovigilance database, from 1967 to 2023 and identified 1508 vaccine-associated uveitis reports among 15,584 uveitis reports recorded for all medicinal products [10]. Reports increased markedly during the COVID-19 pandemic, likely reflecting mass vaccination, increased exposure, and heightened awareness of vaccine-related adverse event reporting [10]. Testi et al. analyzed United Kingdom (UK) Yellow Card data and identified 300 suspected ocular inflammatory events after COVID-19 vaccination, corresponding to 6.6 events per 1,000,000 vaccinated individuals; anterior uveitis was the most commonly reported phenotype, accounting for 58.3% of reports, followed by optic neuritis in 39.3% [21]. Given that pharmacovigilance reports are not always consistently stratified by anatomic subtype, anterior uveitis-specific estimates remain dependent on the U.S. Vaccine Adverse Event Reporting System (VAERS) analyses, cohort studies, and clinically described case series [8,10,11].

To provide a contemporary reporting snapshot, we queried the VAERS, a national passive surveillance system for adverse events following vaccination, on 6 June 2026 and identified 431 reports coded under the Medical Dictionary for Regulatory Activities (MedDRA) preferred term “uveitis” and 74 reports coded as “iridocyclitis,” representing 85.3% and 14.7% of the combined queried reports, respectively [4]. These counts should be interpreted as reporting signals rather than incidence estimates because VAERS is subject to underreporting, stimulated reporting, duplicate entries, incomplete documentation, and variable diagnostic confirmation [22].

### 2.3. Vaccines Implicated

VAAU has been reported after multiple vaccine types, suggesting that post-vaccination anterior uveitis is not restricted to a single antigen, manufacturer, or vaccine platform [6]. Reports have described anterior uveitis after influenza, hepatitis B, human papillomavirus, Bacillus Calmette–Guérin, measles–mumps–rubella, varicella-zoster, yellow fever, COVID-19, and other vaccines [23,24,25,26,27]. Pediatric cases have also been reported following several routine childhood vaccinations, particularly hepatitis B, measles–mumps–rubella (MMR), and varicella vaccines; however, the available evidence is derived largely from spontaneous adverse-event reports and isolated case reports and does not establish a causal relationship [28]. Most non-COVID evidence consists of case reports or small case series and should therefore be interpreted cautiously given the limited level of evidence available [23,24,25].

Pre-COVID-19 reports provide historical context for the range of vaccine exposures associated with anterior uveitis. Cases after yellow fever, Bacillus Calmette–Guérin, measles–mumps–rubella, live attenuated zoster, and other vaccines have described onset intervals ranging from approximately one week to several weeks after vaccination [5,6,25,26,27]. These observations are consistent with a multifactorial model involving vaccine-induced immune stimulation, formulation-specific factors such as adjuvants or delivery systems, and individual host susceptibility [6].

The COVID-19 vaccination era substantially expanded the available clinical literature on VAAU across multiple vaccine platforms [11]. Most published COVID-19 vaccine-associated cases involved messenger RNA (mRNA) vaccines, particularly BNT162b2 and mRNA-1273, likely reflecting their widespread use during mass vaccination campaigns [11]. Reported mRNA-associated cases have included both new-onset anterior uveitis and recurrent inflammatory episodes in patients with prior ocular inflammation [29].

Other COVID-19 vaccine platforms have also been represented in the literature and surveillance datasets. Adenoviral vector vaccines, including ChAdOx1 nCoV-19 and Ad26.COV2.S, have been included in pharmacovigilance and cohort-based analyses of post-vaccination uveitis [20]. Inactivated COVID-19 vaccines have also been associated with isolated ocular inflammatory events, usually with favorable outcomes after anti-inflammatory treatment [30]. Available evidence does not consistently demonstrate that one COVID-19 vaccine platform carries a clearly greater ocular inflammatory risk than another [16].

In the VigiBase analysis, COVID-19 mRNA vaccines showed the strongest disproportionality signal for uveitis reports, followed by hepatitis B, papillomavirus, Ad5-vectored COVID-19, and influenza vaccines [10]. These comparative findings may help identify reporting patterns across vaccine categories, but should not be interpreted as incidence rates or as proof of causality because they may be influenced by reporting volume, vaccine exposure patterns, and stimulated reporting [10].

### 2.4. Temporal Association and Causality

In suspected VAAU, the timing of ocular symptoms is an important component of the clinical assessment but must be interpreted within a broader framework [8]. Symptom onset ranges from hours to several weeks after vaccination, although many cases occur within the first few days to 2 weeks [11,31]. Larger reviews similarly indicate that most ocular symptoms develop within 30 days, typically between 1 and 4 weeks after exposure [5,6,32]. Events beginning within the first week may warrant closer follow-up because earlier presentation has been associated with persistent inflammation or prolonged treatment requirements [5].

Although temporal proximity may raise clinical suspicion, most published studies demonstrate an association in time rather than a confirmed causal relationship between vaccination and anterior uveitis [6,8,11]. Alternative infectious, autoimmune, idiopathic, medication-related, and other non-vaccine-related triggers, as well as spontaneous recurrence or coincidental disease onset, cannot always be completely excluded [6,8,16]. Causal assessment therefore requires careful consideration of competing etiologies, prior uveitis history, recurrence following subsequent vaccine exposure, and expected background rates of anterior uveitis [6,8]. The absence of a disease-specific biomarker further limits the ability to distinguish vaccine-triggered inflammation from coincidental anterior uveitis [6,8].

Controlled evidence remains inconclusive: meta-analytic and matched case–control studies have not demonstrated a significant association between COVID-19 vaccination and uveitis onset, whereas small case series have reported new-onset acute anterior uveitis within early post-vaccination windows, i.e., 2–14 days [14,16,33].

Assessment is further complicated by background uveitis recurrence, previously unrecognized systemic disease, and severe acute respiratory syndrome coronavirus 2 (SARS-CoV-2) infection (i.e., COVID-19), which has been associated with both new-onset and recurrent uveitis [34,35]. These factors should be considered when evaluating suspected vaccine-associated uveitis, particularly in patients with prior uveitis or immune-mediated disease, for whom individualized counseling and monitoring may be more appropriate than vaccine avoidance [20,32]. Underlying autoimmune or inflammatory disease and prior uveitis may alter the baseline risk of disease onset or recurrence [19,20,29,32]. Concurrent or recent infection and medication-related inflammation may also represent competing etiologies that complicate causal attribution [35,36,37,38]. Genetic susceptibility may further modify individual inflammatory risk [7,13,39,40].

## 3. Immunopathogenesis

**Figure 1:**
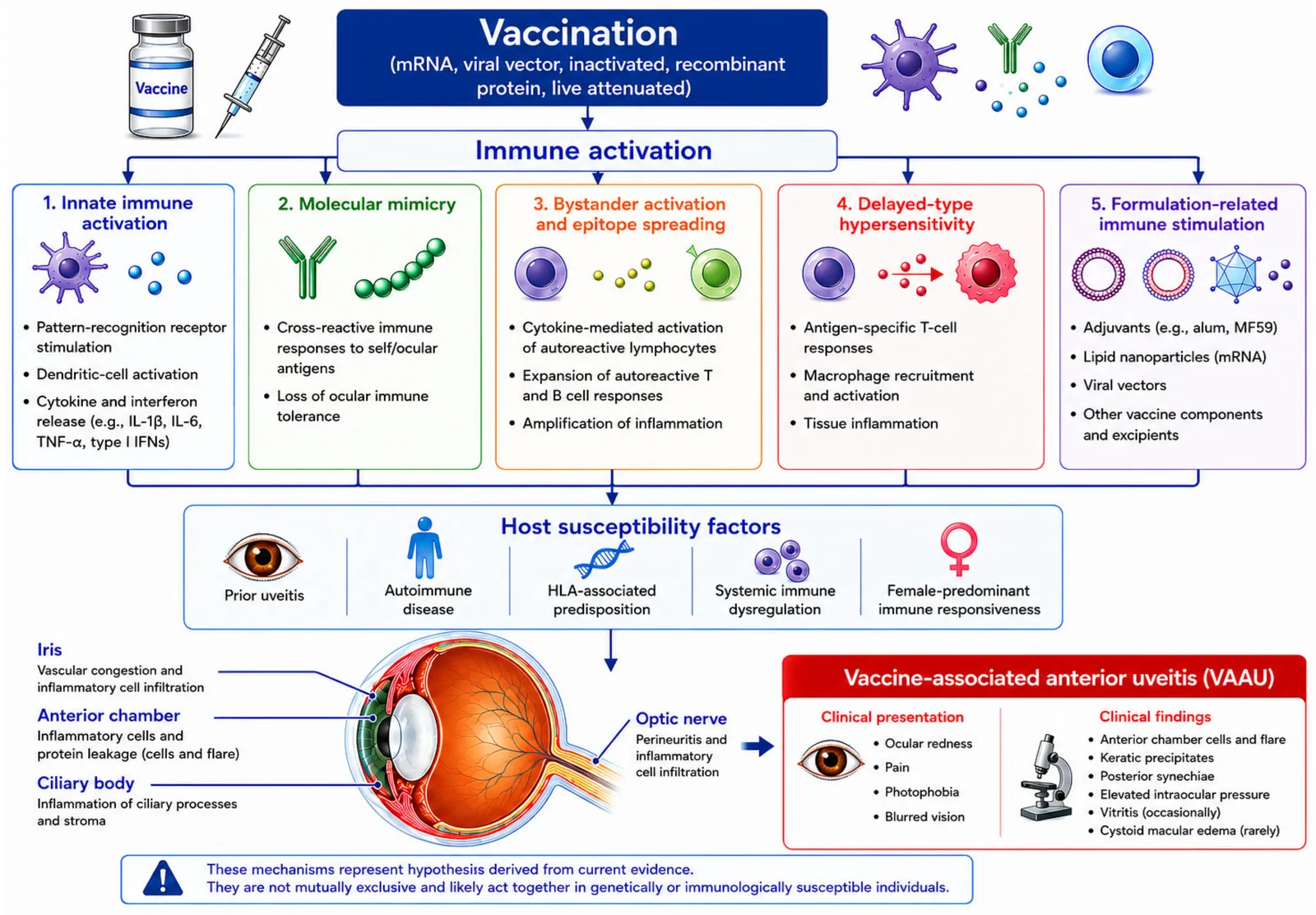
**Proposed immunopathogenic mechanisms underlying VAAU.** Vaccination may trigger immune activation through multiple proposed pathways, including innate immune activation, molecular mimicry, bystander activation and epitope spreading, delayed-type hypersensitivity, and formulation-related immune stimulation. In susceptible individuals, such as those with prior uveitis, autoimmune disease, systemic immune dysregulation, female-predominant immune responsiveness, or HLA-associated genetic predisposition, these mechanisms may contribute to inflammation involving the iris, anterior chamber, and ciliary body, resulting in the clinical phenotype of vaccine-associated anterior uveitis. These proposed mechanisms are not mutually exclusive and may act synergistically in genetically or immunologically susceptible individuals.

The mechanisms underlying VAAU remain incompletely understood and are likely multifactorial [41]. Proposed pathways include innate immune activation, molecular mimicry, delayed-type hypersensitivity, bystander immune activation, immune-complex-mediated inflammation, adjuvant-related responses, polyethylene glycol-related reactions, type I interferon activation, extracellular RNA-mediated vascular effects, nucleic-acid sensing pathways, and immune responses to specific vaccine components [3,33,41]. However, these proposed mechanisms remain largely hypothetical and have not been validated specifically in VAAU through direct mechanistic evidence. The proposed relationships among these mechanisms are summarized in Figure 1.

### 3.1. Innate Immune Activation

Vaccines activate innate immune pathways to initiate protective adaptive immunity [3]. Vaccine antigens, adjuvants, lipid nanoparticles, viral vectors, nucleic acid components, and other formulation-specific components may stimulate pattern-recognition receptors, including toll-like receptors and related innate immune sensors [41]. mRNA vaccines, in particular, can engage endosomal (TLR7/8) and cytosolic (RIG-I/MDA5) nucleic acid sensors, driving type I interferon production and innate immune activation that may lower the threshold for inflammatory responses in predisposed individuals [42]. This activation promotes dendritic-cell maturation, antigen presentation, and release of inflammatory mediators such as interferons and pro-inflammatory cytokines [3]. These effector mediators may disrupt ocular immune homeostasis by increasing vascular permeability, promoting leukocyte recruitment, and amplifying local cytokine signaling within the anterior chamber. Activated dendritic cells, macrophages, and T lymphocytes may then sustain inflammation and contribute to iris and ciliary body tissue injury in susceptible individuals [39,41]. In susceptible individuals, an exaggerated or dysregulated innate response may lower the threshold for ocular inflammation [39].

### 3.2. Molecular Mimicry and Autoimmunity

Molecular mimicry is one proposed pathway by which vaccination could theoretically contribute to autoimmune-like ocular inflammation [43]. This mechanism occurs when immune responses directed against vaccine-related antigens cross-react with structurally similar host antigens [43]. In uveitis, antigenic mimicry has been proposed as a contributor to loss of ocular immune tolerance, although specific cross-reactive targets in VAAU have not been definitively identified [43,44].

### 3.3. Bystander Activation and Epitope Spreading

Bystander activation provides another explanation for post-vaccination ocular inflammation without requiring direct antigenic cross-reactivity [41]. Systemic immune activation after vaccination may stimulate previously quiescent autoreactive lymphocytes through cytokine-rich inflammatory signaling [3]. If inflammation persists, immune responses may broaden through epitope spreading, in which additional self-antigens become immunologic targets [43]. These mechanisms may be particularly relevant in patients with prior uveitis or systemic autoimmune disease, in whom immune tolerance may already be unstable [20].

### 3.4. Delayed-Type Hypersensitivity Reactions

Delayed-type hypersensitivity is mediated primarily by antigen-specific T lymphocytes and macrophage recruitment and typically develops days after antigen exposure [3,44]. The timing of many reported cases and their responsiveness to corticosteroids are consistent with a T-cell-mediated inflammatory process, although these features do not establish causality [29,39].

### 3.5. Host Susceptibility

Host susceptibility likely determines why VAAU develops only in a small subset of vaccinated individuals [41]. A prior history of uveitis appears to be one of the most consistently reported clinical predictors of recurrence following COVID-19 vaccination [20,32,45]. However, this association should not be interpreted as evidence of a direct vaccine-induced effect, as self-controlled studies suggest that the observed increase in post-vaccination episodes may partly reflect the underlying propensity of uveitis to recur in predisposed individuals regardless of vaccination [19]. Systemic autoimmune or inflammatory disease may further lower the threshold for post-vaccination ocular inflammation [29,32,46]. Genetic predisposition, including human leukocyte antigen (HLA)–associated immune responsiveness, may also contribute, although specific HLA associations with VAAU remain insufficiently defined [39].

Female predominance has been observed in vaccine-associated uveitis reports after childhood, suggesting that sex-related immune responsiveness may contribute to susceptibility [10]. However, differences in vaccine exposure, healthcare utilization, and reporting behavior may also influence this pattern [10]. HLA-B27 is a well-established genetic risk factor for acute anterior uveitis [40]. In a multicenter cohort of COVID-19 vaccine-associated ocular inflammation, HLA-B27 positivity was reported in 27.2% of de novo cases and 29.4% of recurrent cases, exceeding expected general-population prevalence [13]. Supporting this view, recent single-cell transcriptomic analyses have identified dendritic cells as key immune populations in HLA-B27-associated acute anterior uveitis, consistent with enhanced local antigen presentation and amplification of inflammatory responses within the anterior chamber [47]. Although this finding does not establish causality, it supports the possibility that HLA-associated immune susceptibility may contribute to post-vaccination ocular inflammation in select patients [13].

## 4. Clinical Presentation, Diagnosis, and Management of VAAU

### 4.1. Clinical Presentation and Spectrum of VAAU

VAAU usually presents with features of acute anterior uveitis, including ocular redness, photophobia, pain, and blurred vision [32,46]. Slit-lamp examination commonly reveals anterior chamber cells and flare, keratic precipitates, and, in some cases, posterior synechiae [32]. The clinical phenotype is variable, with reported cases including non-granulomatous and granulomatous inflammation, unilateral or bilateral involvement, acute de novo presentations, and recurrent or reactivated inflammatory episodes [20,31,32,45].

Clinical severity ranges from mild anterior chamber inflammation with minimal visual impairment to more severe presentations with dense anterior chamber cells, hypopyon, posterior synechiae, elevated intraocular pressure, vitritis, or cystoid macular edema [32]. Sim and Hwang described 11 new-onset acute anterior uveitis cases after COVID-19 vaccination, all with unilateral anterior chamber inflammation and keratic precipitates; several cases showed hypopyon, posterior synechiae, or mild vitritis, and all resolved with corticosteroid-based therapy [33]. Kakil et al. found that anterior uveitis developing after BNT162b2 vaccination was generally milder than idiopathic anterior uveitis, with fewer ocular complications and more favorable visual recovery; symptom onset occurred at a median of approximately 15 days after vaccination, most cases followed the first dose, and no recurrences were observed during 6 months of follow-up [48].

Pediatric post-vaccination ocular inflammation may show a broader anatomic spectrum than many adult anterior-predominant series. Although tertiary referral data suggest that anterior uveitis is the most common anatomic diagnosis among children presenting with uveitis within 28 days of COVID-19 vaccination, intermediate uveitis, posterior uveitis, and panuveitis have also been reported [28,49]. Children may present late, may not reliably report pain, redness, or photophobia, and may already have complications such as posterior synechiae, cataract, glaucoma, band keratopathy, or macular edema at presentation [50]. Compared with many adult series, pediatric post-vaccination ocular inflammatory cases may also require more frequent systemic corticosteroids or increased immunosuppression, especially when underlying inflammatory disease is present [49,50].

Systemic immune status may further influence presentation. Post-vaccination anterior uveitis has been described in the context of systemic immune dysregulation or inflammatory comorbidity [29,32,46]. Rare case reports have described severe or refractory anterior uveitis after vaccination in immunologically complex patients, including allogeneic hematopoietic stem cell transplant recipients with ocular graft-versus-host disease [51].

### 4.2. Diagnostic Approach

**Figure 2:**
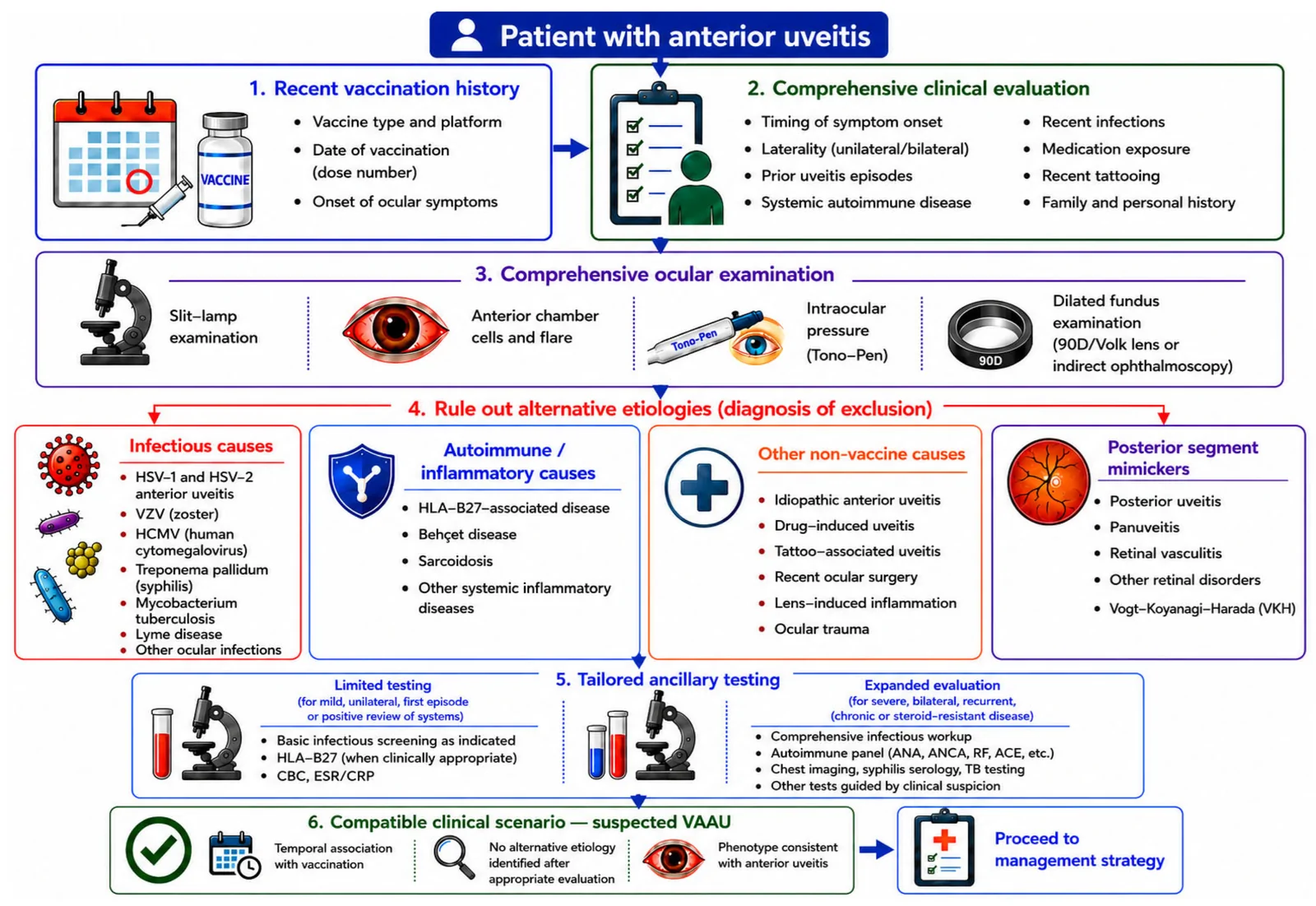
**Diagnostic approach to suspected VAAU.** This framework outlines the stepwise evaluation of anterior uveitis occurring after vaccination. Assessment includes documentation of vaccination history and symptom onset, comprehensive clinical evaluation and ocular examination, and exclusion of infectious, autoimmune, idiopathic, medication-related, postoperative, traumatic, other non-vaccine-related, and posterior-segment causes. Ancillary testing should be tailored according to disease severity, laterality, recurrence, chronicity, and clinical phenotype. VAAU should be considered only when the temporal relationship is compatible, the ocular findings are consistent with anterior uveitis, and alternative etiologies have been reasonably excluded.

Accurate evaluation of suspected VAAU requires careful assessment of temporal association, exclusion of alternative causes of anterior uveitis, and selective use of ancillary testing based on clinical presentation. Suspected VAAU should be considered only after appropriate clinical evaluation has excluded infectious, autoimmune, idiopathic, medication-related, and other established causes of anterior uveitis [6,7]. The overall diagnostic framework is summarized in Figure 2.

#### 4.2.1. Clinical Evaluation

Recent vaccination history should be documented during the evaluation of new anterior uveitis, particularly when symptoms begin within days to weeks after immunization [46]. The clinical history should include vaccine type, date of administration, dose number, laterality and timing of ocular symptoms, prior uveitis history, systemic inflammatory disease, recent infection, medication exposures, recent tattooing, and previous vaccine-associated ocular events. Evaluation of suspected VAAU should include slit-lamp examination, intraocular pressure measurement, and dilated fundus examination to characterize anterior segment inflammation, identify complications, and assess for posterior-segment involvement [7].

#### 4.2.2. Differential Diagnosis

Post-vaccination anterior uveitis should be approached as a diagnosis of exclusion, with attention to infectious reactivation, autoimmune recurrence, idiopathic uveitis, drug-induced inflammation, tattoo-associated uveitis, recent ocular surgery, and posterior-segment inflammatory mimickers [33,45,52].

Infectious uveitis is a particularly important consideration. Viral anterior uveitis, including herpes simplex virus, varicella-zoster virus reactivation, cytomegalovirus reactivation, or herpes zoster ophthalmicus, should be considered when patients present with herpetic skin lesions, keratitis, pigmented or granulomatous keratic precipitates, reduced corneal sensation, elevated intraocular pressure, retinal necrotizing lesions, or recurrent unilateral inflammation [36,37,38]. In such cases, aqueous polymerase chain reaction testing may help distinguish infectious uveitis from presumed immune-mediated VAAU [36,38]. Ott et al. further suggested that a history of herpetic eye disease alone should not be considered a contraindication to COVID-19 vaccination, although individualized counseling may be appropriate for patients with multiple or complex prior episodes [36].

Tuberculous uveitis should be considered in patients from endemic regions or in cases with recurrent, chronic, bilateral, posterior, panuveitic, or treatment-refractory inflammation [34]. Posterior or panuveitic findings should also prompt evaluation for alternative diagnoses, including Vogt–Koyanagi–Harada disease or VKH-like inflammation [31].

#### 4.2.3. Tailored Ancillary Testing

Additional diagnostic evaluation should be tailored to disease severity, laterality, recurrence, chronicity, clinical phenotype, and regional epidemiology rather than applied as a uniform panel to every patient. A first mild, unilateral, non-granulomatous episode in an otherwise healthy patient may require limited testing, whereas severe, bilateral, recurrent, chronic, granulomatous, steroid-resistant, or atypical inflammation warrants broader evaluation for infectious, autoimmune, and masquerade etiologies [7]. When indicated, laboratory testing and ocular imaging should be selected according to the suspected infectious, autoimmune, masquerade, or posterior-segment diagnosis.

### 4.3. Management Strategies and Clinical Outcomes

Management of suspected VAAU should follow established anterior uveitis treatment principles while being individualized according to recurrence, steroid response, anatomic involvement, systemic inflammatory disease, and patient age [7]. Topical corticosteroids are commonly used for anatomically isolated anterior uveitis and should be tapered according to clinical response to reduce rebound inflammation, while cycloplegic agents help reduce pain, relieve ciliary spasm, and prevent or break posterior synechiae [7]. Suspected herpetic or viral anterior uveitis requires antiviral therapy in addition to appropriate anti-inflammatory treatment [36,53].

Escalation should be considered when inflammation is recurrent, steroid-dependent, associated with steroid-induced intraocular pressure elevation, inadequately controlled with topical therapy, involves the posterior segment, or occurs with systemic inflammatory disease [7]. Periocular or systemic corticosteroids may be used when inflammation remains insufficiently controlled with topical therapy and an infectious process has been definitively ruled out [33,54,55]. In pediatric patients with chronic or refractory uveitis, corticosteroids alone may be insufficient, and early steroid-sparing immunomodulatory or biologic therapy may be required to control inflammation and reduce steroid-related complications [49,50].

Clinical outcomes are generally favorable with appropriate anti-inflammatory therapy. In published series, most eyes achieved quiescence without lasting ocular morbidity or permanent vision loss after observation, topical corticosteroids, periocular corticosteroids, or systemic corticosteroids when indicated [13,33,48].

Persistent visual loss appears uncommon and is more often related to pre-existing ocular disease, delayed treatment, recurrent inflammation, or complications such as cystoid macular edema, cataract, glaucoma, or posterior synechiae than to uncomplicated VAAU itself [7,13,31,33]. In the pediatric multinational case series by Testi et al., all patients had final visual acuity unaffected or decreased by less than three lines, with complications occurring mainly in children with underlying inflammatory disease such as Behçet’s disease or juvenile idiopathic arthritis [49]. Yiu et al. reported that delayed diagnosis, chronic relapsing disease, steroid response, glaucoma, cataract, posterior synechiae, and macular edema may worsen pediatric prognosis [50]. Zou et al. found that persistent inflammation or treatment duration of greater than 4 weeks may occur in a subset of non-COVID antiviral vaccine-associated ocular inflammatory events, particularly when inflammation is severe, retinal or posterior involvement is present, or symptoms begin very early after vaccination [5].

## 5. Discussion and Broader Implications

### 5.1. Interpretation of the Current Evidence

The available literature supports VAAU as an uncommon, clinically relevant, and usually treatment-responsive ocular inflammatory phenotype. However, the current evidence does not establish a uniform vaccine-induced disease. Suspected VAAU should instead be interpreted as a diagnosis of exclusion requiring assessment of the clinical phenotype, timing, competing etiologies, and background risk of uveitis [6,7]. This framework allows clinicians to recognize possible post-vaccination inflammation while avoiding inappropriate attribution of coincidental anterior uveitis to vaccination.

VAAU also appears clinically heterogeneous. Habot-Wilner et al. described both de novo and recurrent COVID-19 vaccine-associated uveitis presentations, supporting variability in clinical phenotype [29]. Many reported cases respond to conventional anti-inflammatory therapy, whereas recurrent, severe, or treatment-intensive courses have been described in selected groups, including patients with prior uveitis, pediatric patients, and immunologically complex individuals [20,49]. These patterns support a model in which vaccination may act as a nonspecific immune stimulus in predisposed hosts rather than as an independent cause of anterior uveitis in most vaccinated persons [20].

### 5.2. Clinical Counseling and Vaccine-Safety Implications

The potential risk of VAAU should be interpreted within the broader context of vaccine benefit [56]. Vaccines should be evaluated not only for rare temporally associated ocular adverse events, but also for their protective role against ocular morbidity caused by vaccine-preventable infections [56]. Given the eye’s immune-privileged status, certain infections may cause disproportionate tissue injury once ocular barriers are breached [56].

Available evidence generally supports the ocular safety profile of vaccination while reinforcing the importance of continued surveillance. Controlled studies have not consistently demonstrated an increased risk of anterior uveitis after vaccination, and reported ocular inflammatory events remain rare relative to the scale of immunization programs [14,16,21]. Additional evidence suggests that vaccination may reduce infection-associated inflammatory risk in susceptible populations by decreasing the burden of vaccine-preventable disease and related immune activation [35,57].

In terms of clinical counseling, patients should be advised to seek ophthalmic evaluation if they develop ocular redness, pain, photophobia, or blurred vision after vaccination, while being reassured that most reported cases are treatable and that rare reports of ocular inflammation do not justify vaccine avoidance [32,58]. Individualized counseling is most relevant for patients with prior ocular inflammation, autoimmune disease, previous vaccine-associated ocular events, immunosuppression, or other susceptibilities. These patients may benefit from closer ophthalmic monitoring after vaccination, particularly during the first month [5,41]. Limited observational evidence also suggests that continuation of systemic corticosteroid therapy in these select patients at the time of COVID-19 vaccination may be associated with a lower risk of post-vaccination uveitis relapse, although this finding requires further validation [59].

Post-marketing safety systems remain essential for identifying rare ocular inflammatory signals after vaccination [6]. However, as emphasized by Cheng and Margo and by Oh et al., passive reports should be interpreted as hypothesis-generating rather than definitive because they are limited by underreporting, stimulated reporting, incomplete clinical documentation, reporting bias, and lack of standardized ophthalmic confirmation [6,10]. Thus, suspected VAAU reports should support continued surveillance and clinical awareness [6].

### 5.3. Limitations of the Available Evidence

The generalizability of the available evidence is limited by the rarity of reported VAAU and the predominance of case reports, small case series, selected cohorts, and single-institution studies [6,10,14]. These study designs may not capture the full clinical spectrum of anterior uveitis in the broader vaccinated population. In addition, reliance on passive surveillance systems introduces underreporting, stimulated reporting, incomplete clinical documentation, duplicate reports, and variable diagnostic confirmation. The frequent inclusion of anterior uveitis within broader uveitis datasets also limits phenotype-specific incidence estimation and causal interpretation [6,8,10,22].

### 5.4. Future Directions

Future research should move beyond passive reporting systems and isolated case reports toward prospective, standardized investigations of VAAU. Population-based cohorts, active surveillance registries, and harmonized case definitions following established criteria such as those defined by the Standardization of Uveitis Nomenclature (SUN) working group are needed to better characterize phenotype, incidence, recurrence risk, clinical severity, treatment response, and long-term visual outcomes [6,14,60,61]. Future studies should also validate pharmacovigilance signals using population-based cohorts, dose–response analyses, severity-stratified outcomes, and adjustment for prior infection, ankylosing spondylitis, autoimmune disease, immunosuppression, and other established uveitis risk factors [14].

Studies to clarify whether reported cases reflect true vaccine-triggered inflammation, coincidental disease onset, or interaction between vaccination and pre-existing immune susceptibility are also necessary [41]. Advanced approaches, including aqueous humor cytokine profiling, immune-cell characterization, HLA analysis, and molecular biomarker discovery, may help identify patients at increased risk and improve understanding of disease pathogenesis [39,41]. Comparative studies across vaccine platforms and investigations incorporating prior infection status, systemic inflammatory disease, and uveitis history may further refine attribution.

Lastly, standardized reporting is needed to improve comparability across studies. Future reports should consistently document vaccine platform, dose number, timing of symptom onset, anatomic classification, disease severity, prior uveitis history, systemic comorbidities, diagnostic evaluation, treatment, recurrence status, and visual outcomes to facilitate future meta-analyses and strengthen interpretation of vaccine-safety signals.

## 6. Conclusions

Vaccine-associated anterior uveitis is an uncommon, phenotype-specific ocular inflammatory event reported after multiple vaccine platforms. Current evidence primarily supports a temporal association and does not definitively establish causality. Its diagnosis therefore requires careful distinction among coincidental disease onset, background uveitis occurrence or recurrence, and potentially vaccine-triggered inflammation. Most described cases are acute, treatment-responsive, and associated with favorable visual outcomes, although recurrent or more severe inflammation may occur in patients with prior uveitis or underlying immune-mediated disease. Given the substantial individual and public-health benefits of immunization, isolated reports of VAAU should not discourage vaccination when otherwise clinically indicated. Continued surveillance, rigorous epidemiologic studies, mechanistic investigation, and individualized counseling for higher-risk patients are needed to refine risk assessment and improve care for this uncommon clinical entity.

## Data Availability

No new data were created or analyzed in this study. Data sharing is not applicable to this article.

## Abbreviations

ANA	Antinuclear antibody
ANCA	Antineutrophil cytoplasmic antibody
CI	Confidence interval
COVID-19	Coronavirus disease 2019
HLA	Human leukocyte antigen
HLA-B27	Human leukocyte antigen B27
MedDRA	Medical Dictionary for Regulatory Activities
mRNA	Messenger RNA
OR	Odds ratio
RNA	Ribonucleic acid
SARS-CoV-2	Severe acute respiratory syndrome coronavirus 2
TB	Tuberculosis
UK	United Kingdom
VAAU	Vaccine-associated anterior uveitis
VAERS	Vaccine Adverse Event Reporting System
VKH	Vogt–Koyanagi–Harada
WHO	World Health Organization
